# Molecular epidemiology of *Plasmodium *species prevalent in Yemen based on 18 s rRNA

**DOI:** 10.1186/1756-3305-3-110

**Published:** 2010-11-19

**Authors:** Abdulsalam MQ Al-Mekhlafi, Mohammed AK Mahdy, Ahmed A Azazy, Mun Yik Fong

**Affiliations:** 1 Department of Parasitology, Faculty of Medicine, University of Malaya, 50603 Kuala Lumpur, Malaysia; 2 Department of Parasitology, Faculty of Medicine and Health Sciences, Sana'a University, Sana'a - Yemen

## Abstract

**Background:**

Malaria is an endemic disease in Yemen and is responsible for 4.9 deaths per 100,000 population per year and 43,000 disability adjusted life years lost. Although malaria in Yemen is caused mainly by *Plasmodium falciparum *and *Plasmodium vivax*, there are no sequence data available on the two species. This study was conducted to investigate the distribution of the *Plasmodium *species based on the molecular detection and to study the molecular phylogeny of these parasites.

**Methods:**

Blood samples from 511 febrile patients were collected and a partial region of the 18 s ribosomal RNA (18 s rRNA) gene was amplified using nested PCR. From the 86 positive blood samples, 13 *Plasmodium falciparum *and 4 *Plasmodium vivax *were selected and underwent cloning and, subsequently, sequencing and the sequences were subjected to phylogenetic analysis using the neighbor-joining and maximum parsimony methods.

**Results:**

Malaria was detected by PCR in 86 samples (16.8%). The majority of the single infections were caused by *P. falciparum *(80.3%), followed by *P. vivax *(5.8%). Mixed infection rates of *P. falciparum *+ *P. vivax *and *P. falciparum *+ *P. malariae *were 11.6% and 2.3%, respectively. All *P. falciparum *isolates were grouped with the strain 3D7, while *P. vivax *isolates were grouped with the strain Salvador1. Phylogenetic trees based on 18 s rRNA placed the *P. falciparum *isolates into three sub-clusters and *P. vivax *into one cluster. Sequence alignment analysis showed 5-14.8% SNP in the partial sequences of the 18 s rRNA of *P. falciparum*.

**Conclusions:**

Although *P. falciparum *is predominant, *P. vivax*, *P. malariae *and mixed infections are more prevalent than has been revealed by microscopy. This overlooked distribution should be considered by malaria control strategy makers. The genetic polymorphisms warrant further investigation.

## Background

Malaria still continues to be a devastating global public health problem in more than 100 countries with 3.2 billion people being at risk [1]. Of this number, 300-500 million people contract the disease each year, resulting in 2-3 million deaths [2]. This includes 1 million children of less than five years of age [3].

The genus *Plasmodium *consists of nearly 200 species that infect humans, birds, reptiles and mammals. It belongs to the phylum Apicomplexa. Five *Plasmodium *species have been known to infect humans: *P. falciparum, P. vivax, P. malariae, P. ovale*, and *P. knowlesi *[4]. Much work on molecular phylogeny has focused on the relationship between *Plasmodium *species and the origin of human malaria [5-7]. Although the 18 s rRNA gene appears to be robust for phylogenetic analysis, its sequences should be analysed carefully since the gene could be expressed as one of the three types during the different developmental stages of the malaria parasite [4,8]. Asexual type (A) and sporozoite type (S) were found in *P. falciparum*, *P. berghei*, *P. vivax*, and other species [4,9] while oocyst type (O) has been reported in *P. vivax *[10].

Yemen is a country in the East Mediterranean region where the highest incidence of malaria has been registered after Afghanistan [11] and is responsible for 4.9 deaths per 100000 per year and 43000 disability adjusted life years (DALYs) lost [12]. Giemsa microscopy is the most common diagnostic tool used in this country. This technique has drawbacks, however, in terms of detecting low parasitemic cases and the proper identification of mixed infection could actually underestimate the malaria situation in Yemen. Thus, the molecular approach was applied for the first time, in this country, to investigate the molecular epidemiology of malaria. The study will indicate the magnitude of malaria co-infection which should be considered in clinical case management. In addition, malaria strains, genetic polymorphism and the molecular phylogeny of Yemen *Plasmodium *isolates will be studied.

## Methods

### Study area

The present study was conducted in five governorates with a total population of 7.9 million[13]. The selected governorates included Taiz and Hodeidah, which represent the mountainous hinterland and coastal areas, respectively; and Rymah, Dhamar and Sana'a from the highland areas. Most houses, especially in the rural areas, have a wooden roof. Occupations include agriculture, fishing, livestock and handicrafts. The peak time of malaria transmission in the coastal areas occurs in winter (October-April), while in the western mountains, the peak occurs in the summer (May-September). In the highland areas, which are located at more than 2000 metres above sea level, the transmission of malaria occurs throughout the year [14]. *Anopheles arabiensis *is the main vector in the country but *Anopheles culicifacies *plays an important role in the transmission of malaria in the coastal area. *Anopheles sergenti *has also been reported in the mountainous hinterland and highland areas [14]. There is a paucity of comparative data on mortality and morbidity caused by malaria in the three malaria endemic areas. An observational study showed that mortality rate among children with severe malaria was 2.4% and 3.5% for costal and hinterlands, respectively [15]. However, no data were available on mortality rate in the highland areas.

### Sample collection and genomic DNA preparation

A total of 511 finger-prick blood samples were collected from symptomatic patients attending hospitals or medical centres for the treatment of malaria. Blood from finger pricks from febrile patients was collected and spotted on Whatman 3 MM filter papers (Whatman International Ltd., Maidstone, England) and slides to prepare thin and thick blood films. Each blood spot on Whatman filter paper was allowed to air-dry and was stored in a separate sealed plastic bag at room temperature until DNA extraction was required. Parasite genomic DNA was extracted from the blood collected on filter paper using QIAgen DNA Mini Kit blood and tissue (QIAGEN, cat. no. 51306, Germany) according to the manufacturer's instructions. Briefly, a disc was punched out from the blood spot using a pre-flamed paper puncher and placed in 1.5 ml centrifuge tubes using clean, flamed forceps.

### Polymerase chain reaction (PCR)

Genus-and species-specific nested PCR assays based on the 18 s rRNA gene were used to detect and identify *Plasmodium *species as previously reported [16]. Primary PCR was carried out using genus-specific primers (rPLU1 and rPLU5). PCR reaction was run in a total of 25 μL reaction mixture containing 4 μL genomic DNA, 1× i-Taq™buffer including MgCl_2 _(iNtRON BIOTECHNOLOGY, Seoul, Korea), 250 μM dNTP (iNtRON BIOTECHNOLOGY, Seoul, Korea)), 200 nM of each primer and 1.25 U of i-Taq™DNA polymerase (iNtRON BIOTECHNOLOGY, Seoul, Korea). Secondary PCR was carried out using species-specific primers (rFAL1/rFAL2, rVIV1/rVIV2, rMAL1/rMAL2 and rOVA1/rOVA2); each primer pair was placed in a single tube. PCR mixtures were as mentioned above, except that 2 μL of the first PCR product was used as a template in the secondary PCR.

The cycling conditions for primary PCR were as follows: an initial denaturation step at 95°C for 10 minutes, then 40 cycles at 94°C for 20 seconds, annealing at 55°C for 20 seconds, extension at 72°C for 1 minute, and a final extension at 72°C for 5 minutes. The reaction was terminated by reducing the product temperature to 10°C. PCR products were stored at -20°C until analysis. Secondary PCR used similar cycling conditions except that the number of cycles was reduced to 35 cycles.

### Cloning and DNA sequencing

The PCR product comprising 17 positive samples (13 *P. falciparum *and 4 *P. vivax*), representing different geographical areas, was selected for cloning and subsequent sequencing. A partial sequence (~1200 bp) of the 18 s rRNA gene was amplified with primers rPLU6 and rPLU5. PCR was carried out in a volume of 20 μl mixture containing 1× Phusion HF buffer (Finnzymes, Finland), 200 μM of each dNTP (Finnzymes) and 400 nM of each primer, and 1 U of Phusion DNA Polymerase (Finnzymes). The PCR conditions were as follows: initial denaturation at 98°C for 30 seconds, followed by 40 cycles of amplification at 98°C for 7 seconds, 59°C for 20 seconds and 72°C for 48 seconds followed by a final extension step at 72 °C for 5 minutes. The amplified products were cloned into Zero Blunt^® ^vector according to the manufacturer's instructions (cat. No. K2700-20; Invitrogen, USA). At least 20 colonies from each of transformation reactions were screened using the rPLU6 and rPLU5 primers. Amplification was done in a 20 μl reaction mixture containing 1× reaction buffer (5× Green Go Taq Flexi Buffer, Promega Madison USA), 2 mM MgCl_2 _(Promega), 200 mM of each dNTP (Promega), 300 nM of each primer and 0.5 U Go Taq DNA polymerase (Promega,). PCR conditions were as follows: initial denaturation of 94°C for 10 minutes, followed by 30 cycles of amplification at 94°C at 45 seconds, annealing at 55°C at 1 minute, extension at 72°C for 1 minute 30 seconds, followed by a final extension step at 72°C at 10 minutes. Plasmids from clones having the correct insert were extracted using QIAprep^® ^Spin Miniprep kit (QIAgen, cat. no. 27106, Germany) following the manufacturer's instructions. Purified plasmid containing the insert was sequenced in both directions using the ABI PRISM^® ^BigDye^™ ^terminator v3.0 Ready Reaction Cycle Sequencing Kit (Applied Biosystems, USA) in an 3700 DNA Analyzer (Applied Biosystems, USA).

### Phylogenetic analysis

DNA sequences (forward and reverse) were edited and the consensus sequence was created using the BioEdit. Consensus sequences were multiple-aligned with previously published sequences from the GenBank database using MEGA4 software http://www.megasoftware.net. Phylogenetic analysis was performed with the MEGA4 software. Two types of phylogenetic analysis were used on the aligned sequences to assess relationships among isolates; a distance-based Neighbor-Joining (NJ) analysis was performed calculated with the Kimura 2-parameter [17], and Maximum Parsimony (MP) analysis was performed using the Close-Neighbour-Interchange algorithm [18]. The reliability of the trees was assessed by the bootstrap method with 1,000 replications [19]. Similarity searches were carried out using the Basic Local Alignment Search Tool (BLAST) [20].

## Results

A total of 511 blood samples from febrile patients were screened by using 18 s rRNA-based nested PCR. Of the 511 samples, 86 (16.8%) were positive for malaria. A majority of the malarial infections were due to *P. falciparum *(80.3%). *Plasmodium vivax *infections were seen in only 5.8% of the samples. Double infections with *P. falciparum *+ *P. vivax *(11.6%) and *P. falciparum *+ *P. malariae *(2.3%) were also detected (Table 1). The PCR product of 13 *P. falciparum *isolates and four of *P. vivax *were selected from different geographical areas including the coastal area, the hinterland and the highland malaria endemic areas, and subjected to cloning and sequencing. The 17 sequences representing *P. falciparum *and *P. vivax *from this study (Table 2) and 13 sequences representing human, bird and primate malaria from the GenBank database, were multiple aligned and analysed using the Neighbor-Joining (NJ) and Maximum Parsimony (MP) methods.

**Table 1:** Distribution of malaria species in the study population

Variable	Percentage
**Species**	
*P. falciparum*	69 (80.3%)
*P. vivax*	5 (5.8%)
*P. falciparum *&*P. vivax*	10 (11.6%)
*P. falciparum *&*P. malariae*	2 (2.3%)

**Table 2:** Yemen isolates of *P. falciparum *and *P. vivax *used in the phylogenetic analysis

SN	ID	Species	Geographical areas	**GenBank Accession No**.
3	D122	*P. falciparum*	Dhamar	HQ283210
6	D124	*P. falciparum*	Dhamar	HQ283211
11	H12	*P. falciparum*	Hodiedah	HQ283212
2	H61	*P. falciparum*	Hodiedah	HQ283213
13	H87	*P. falciparum*	Hodiedah	HQ283214
10	R73	*P. falciparum*	Raymah	HQ283215
9	R117	*P. falciparum*	Raymah	HQ283216
5	T71	*P. falciparum*	Taiz	HQ283217
7	T91	*P. falciparum*	Taiz	HQ283218
4	T113	*P. falciparum*	Taiz	HQ283219
1	T116	*P. falciparum*	Taiz	HQ283220
8	T122	*P. falciparum*	Taiz	HQ283221
12	T124	*P. falciparum*	Taiz	HQ283222
17	D24	*P. vivax*	Dhamar	HQ283223
14	H15	*P. vivax*	Hodiedah	HQ283224
16	H108	*P. vivax*	Hodiedah	HQ283225
15	H120	*P. vivax*	Hodiedah	HQ283226

**Figure 1:**
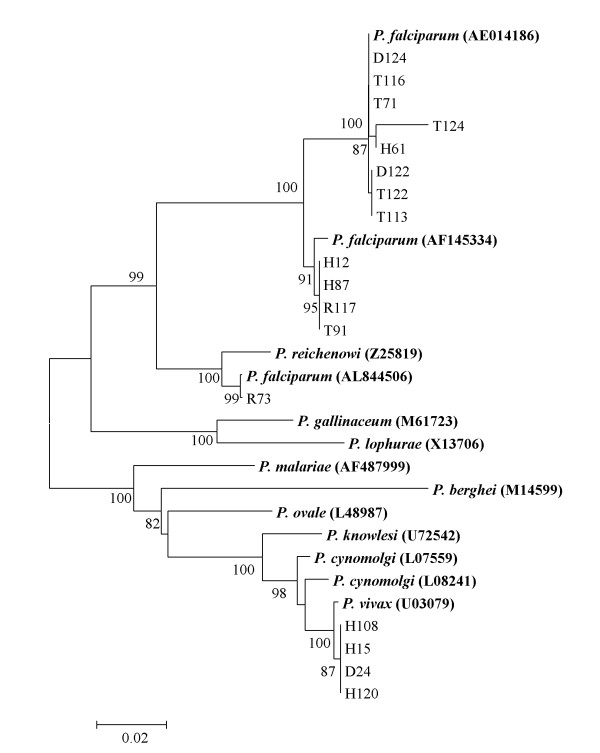
**Neighbor-Joining (NJ) tree, constructed based on the nucleotide sequences of 18 s rRNA, displaying the relationships of 17 sequences representing 13 *P. Falciparum *isolates and 4 sequences representing 4 *P. Vivax *isolates**. Bootstrap support of more than 80% is indicated. Bold-type represents reference sequences for *Plasmodium *species from GenBank.

The NJ method (Figure 1) shows a main cluster for *P. falciparum *and *P. reichenowi*, the chimpanzee malaria (100 bootstrap). Within this main cluster, the Yemen *P. facliparum *isolates were placed into three sub-clusters. The first sub-cluster includes 8 isolates which grouped with *P. falciparum *3D7 (GenBank Accession number AE014186) (100 bootstrap). The second sub-cluster comprises 4 Yemen isolates and *P. falciparum *Papua New Guinea (GenBank Accession number AF145334) (100 bootstrap). The Yemen *P. falciparum *isolate R73 diverged from other isolates and clustered with *P. falciparum *(GenBank Accession number AL844506) (99 bootstrap). The four Yemen isolates of *P. vivax *were grouped in one cluster with *P. vivax *Salvador1 (GenBank Accession number U03079) (100 bootstrap). The monkey malaria parasites (*P. cynomolgi *and *P. knowlesi*) were grouped with human *P. vivax *in one cluster (100 bootstrap). Bird malaria parasites (*P. gallinaceum *and *P. lophurae*) formed a separate cluster (100 bootstrap). The maximum parsimony tree (Additional file 1) is concordant in topology with the NJ tree.

The thirteen 18 s rRNA sequences of Yemen isolates of *P. falciparum *were multiple aligned against the 1135-nucleotide sequence of 18 s rRNA of *P. falciparum *3D7 (GenBank Accession number AE014186) to investigate the genetic diversity of *P. falciparum *in Yemen (Additional file 2). The isolate T124 showed 25 SNP (19 substitutions, 1 insertion and 5 deletions). Isolates H61 and D122 showed 6 SNP (1 substitution and 5 deletions) and 8 SNP (1 substitution, 4 insertions and 3 deletions), respectively. Isolates T122 and T113 showed 7 SNP (1 substitution, 1 insertion and 5 deletions). Isolates D124, T116 and T71 had 10 SNP (10 deletions). Isolates R117, H12, T91 and H87, which were clustered together in the phylogenetic analysis, showed polymorphism in 5% of the 1135-nucleatide sequence. The highest polymorphism (14.8%) was noted in the isolate R73 which diverged in a separated cluster in the phylogenetic trees. The four sequences of the Yemen isolates of *P. vivax *were also aligned against 1058-nucleotide sequence of *P. vivax *Salvador1 (GenBank Accession number U03079). The isolate H108 had 7 SNP (6 substitutions and 1 insertion). The isolate H15 had 7 SNP (5 substitutions and 2 insertions). The isolates D24 and H124 showed 5 SNP (5 substitutions).

**Figure 2:**
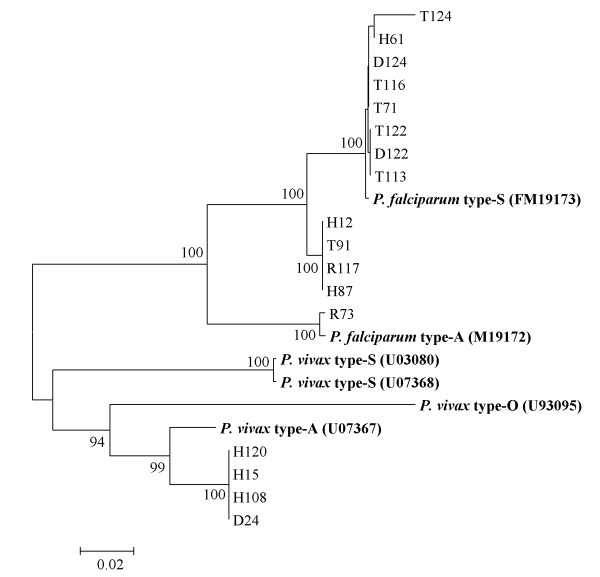
**Neighbor-Joining (NJ) tree, constructed based on the nucleotide sequences representing the different types of 18 s rRNA (A, O or S type) from GeneBank and 13 sequences representing 13 *P. falciparum *isolates and 4 sequences representing 4 *P. Vivax *isolates from this study**. Bootstrap support of more than 90% is indicated. Bold-type represents reference sequences for *Plasmodium *species from GenBank.

To determine whether the genetic diversity is due to the heterogeneous types of *Plasmodium *18 s rRNA or not, phylogenetic trees were re-constructed based on our sequences and sequences representing different types of *Plasmodium *18 s rRNA (A, O or S type) from the GenBank database. Both the NJ tree (Figure 2) and MP tree (Additional file 3) were consistent, placing all the Yemen isolates of *P. falciparum *except isolate R73 into one cluster and clade with *P. falciparum *type-S (GenBank Accession number M19173) (100 bootstrap). The isolate R73 was much closer to *P. falciparum *18 s rRNA type-A (GenBank Accession number M19173) (100 bootstrap). All *P. vivax *isolates were grouped with *P. vivax *type-A in one cluster (GenBank Accession number U07367) (100 bootstrap).

## Discussion

This is the first study to apply molecular techniques to study the epidemiology and phylogeny of malaria in Yemen. Malaria infection rate, using 18 s rRNA-based nested PCR, was 16.8% which is comparable with previous studies carried out in Yemen based on Giemsa microscopy [15,21-25]. PCR results showed that approximately 12% of the positive malaria cases were mixed infection of *P. falciparum *and *P. vivax *or *P. malariae *which is a higher rate than has been hitherto reported. The high proportion of mixed infection using molecular identification could be explained by the high sensitivity of PCR [26] and the low level parasitaemia of the dominated species which is under the diagnostic threshold of Giemsa microscopy. Accurate identification of mixed infection with malaria species is very important for proper clinical case management. Malaria treatment policy differs depending on the infecting species. Thus, in the case of mixed infection, if only one species is treated, the other may establish a new episode of malaria.

*Plasmodium falciparum *is the dominant malaria species in Yemen followed by *P. vivax*, whereas *P. malariae *has rarely been reported in Yemen. The current study highlighted the possibility that *P. malariae *is distributed more than expected and it is often overlooked due to low levels of parasitaemia and the shortcoming of microscopy, the only technique used for malaria detection and identification in the country. Furthermore, *P. malariae *may be obscured by the dominant species. The underestimation of *P. malariae *distribution has been reported in other countries [27-30]

Phylogenetic analysis using the NJ method grouped Yemen isolates of *P. falciparum *into one main cluster. Within the main cluster, the Yemen isolates were placed in three sub-clusters (Figure 1). These clusters were supported by cladograms using the MP method. One sub-cluster contained 8 isolates and *P. falciparum *3D7 (GenBank Accession number AE014186) [31]. The second sub-cluster included four isolates and *P. falciparum *(GenBank Accession number AF145334) which was isolated from Papua New Guinea [32]. The isolate R73 formed a separate cluster with *P. falciparum *(GenBank Accession number AL844506). Phylogenetic analysis based on our sequences and sequences from the GenBank representing the secondary structure of 18 s rRNA showed that isolate R73 that diverged in a separate cluster may be A-type ribosomal rRNA, while the other two sub-clusters are S-type rRNA. Thus, genetic diversity within the Yemen isolates that grouped in one cluster with the S-type rRNA may be not due to the heterogeneity of 18 s rRNA genes. Genetic diversity may provide a mechanism for drug resistance which may affect any intervention strategy based on treatment. Furthermore, PCR-based specific diagnosis of *Plasmodium *species may fail due to sequence variations in the priming regions, thus leading to false negative results. The low sensitivity of PCR-based diagnosis due to genetic mutations has been previously reported [30].

## Conclusions

In conclusion, malaria is a major public health problem in Yemen. Although *P. falciparum *is predominant, *P. vivax*, *P. malariae *and mixed infections are more prevalent than has been revealed by microscopy. This overlooked distribution should be considered by malaria control strategy makers. Sequences from *P. falciparum *isolates showed high genetic polymorphisms that may not be related to the variants of ribosomal RNA expressed in the different stages of malaria parasites which warrant further studies.

## Competing interests

The authors declare that they have no competing interests.

## Authors' contributions

AMQA, MAKM, FMY and AAA and designed the study; AMQA dealt with study subjects in the field, carried out the laboratory work and collected the data; AMQA and MAKM performed the statistical analysis; AMQA, MAKM, FMY and AAA and interpreted the data; AMQA and MAKM drafted the manuscript; AMQA, FMY, AAA, MAKM contributed to the revision of the manuscript. All authors read and approved the final manuscript. AMQA and FMY are guarantors of the paper.

## Supplementary Material

Additional file 1 **Maximum Parsimony (MP) tree **. The tree was constructed based on the nucleotide sequences of 18 s rRNA, displaying the relationship of 17 sequences representing 13 *P. falciparum *isolates and 4 sequences representing 4 *P. vivax *isolates. Bootstrap support of more than 60% is indicated. Bold-type represents reference sequences for *Plasmodium *species from GenBank. Click here for file

Additional file 2 **Multiple alignment of partial 18 s rRNA sequences**. Multiple alignment of partial 18 s rRNA sequences of Yemen *Plasmodium falciparum *isolates against *P. falciparum *3D7 (GenBank accession numbers AE014186). Dots represent sequence identities. Dashes denote no nucleotides. Click here for file

Additional file 3 **Maximum parsimony (MP) tree**. The tree was constructed based on nucleotide sequences representing the different types of 18 s rRNA (A, O or S type) from GeneBank and 13 sequences representing 13 *P. falciparum *isolates and 4 sequences representing 4 *P. vivax *isolates from this study. Bootstrap support of more than 90% is indicated. Bold-type represents reference sequences for *Plasmodium *species from GenBank. Click here for file
